# Identification of two potential inhibitors of *Sporothrix brasiliensis* and *Sporothrix schenckii* in the Pathogen Box collection

**DOI:** 10.1371/journal.pone.0240658

**Published:** 2020-10-14

**Authors:** Luana Pereira Borba-Santos, Taissa Vila, Sonia Rozental

**Affiliations:** Instituto de Biofísica Carlos Chagas Filho, Universidade Federal do Rio de Janeiro, Rio de Janeiro, Rio de Janeiro, Brazil; University of Tsukuba, JAPAN

## Abstract

Sporotrichosis is a neglected endemic mycosis with a high incidence in Latin America, mainly in Brazil. *Sporothrix schenckii* is the most frequent species in Latin America, whereas *Sporothrix brasiliensis* is the predominant species observed in Brazil and is associated with both human and animal sporotrichosis. Sporotrichosis treatment remains restricted to a few options, itraconazole being the first choice for human and animal therapy. In this work, we screened the molecular library Pathogen Box (Medicines for Malaria Venture [MMV], Switzerland) in search of compounds with anti-*Sporothrix* activity. Our initial screen of the 400 compounds identified five compounds that inhibited more than 80% of *S*. *brasiliensis* and *S*. *schenkii* growth. Among those, three compounds (MMV675968, MMV102872, and MMV002817 (known as iodoquinol)) not previously described as antifungals or agrochemicals, were selected for further evaluation. MMV102872 and iodoquinol showed the most promising combination of antifungal activity (lower inhibitory concentration) and fungal selectivity (lower cytotoxicity in LLC-MK2 cells). Scanning electron microscopy and flow cytometry analyses revealed that MMV102872 and iodoquinol induced changes in cell morphology, membrane integrity, and the presence of neutral lipids, impairing fungal survival. Our results indicate that MMV102872 and iodoquinol are promising molecules for use as scaffolds for the development of new antifungal agents.

## Introduction

Sporotrichosis is a neglected endemic mycosis caused by thermodimorphic fungi of the genus *Sporothrix* that affects humans and animals [1]. In Brazil, sporothrichosis is an endemic disease and an important zoonosis, with thousands of humans and cats being infected in the last two decades [2]. Between 1992 and 2015, 782 hospitalizations and 65 deaths due to human sporotrichosis were reported in Brazil [3], and outbreaks of the disease are currently present in areas of the southeast, south, and northeast of the country [4].

*Sporothrix schenckii* and *Sporothrix brasiliensis* are the most virulent species in the *Sporothrix* genus and the main etiological agents of sporotrichosis in Latin America and Brazil, respectively [4, 5]. Infection with *S*. *schenckii* occurs by traumatic inoculation of fungus present in contaminated organic materials, while *S*. *brasiliensis* transmission is associated with the zoonotic route involving cats as intermediate hosts [6]. Importantly, *S*. *brasiliensis* can induce a pronounced inflammatory response, leading to more severe and/or atypical clinical presentations of sporotrichosis [7, 8].

Although sporotrichosis is endemic in several countries, treatment remains restricted to a few options, relying mainly on itraconazole [4]. The emergence of clinical and feline-borne isolates with low *in vitro* susceptibility to itraconazole and the constantly reported therapeutic failure highlights the need to seek new options for sporotrichosis treatment [9–11].

High-content screening of libraries containing hundreds of drug-like compounds is a powerful strategy to search for novel antifungal drugs and to explore new targets [12]. Screening of large chemical libraries aim to identify compounds able inhibit the growth of pathogenic fungi and have been the most successful approach in the discovery of chemical scaffolds with promising antifungal action [12]. However, good quality of compounds and the establishment of standardized assays for susceptibility tests are required for this approach to succeed [12].

The Pathogen Box library, developed by the Medicines for Malaria Venture organization (MMV), contains 400 compounds with repurposing potential and also new molecules (https://www.mmv.org/mmv-open/pathogen-box), selected on the basis of their promising activity against pathogens associated with neglected tropical diseases [13]. In an effort to identify new molecules with antifungal activity, the Pathogen Box library was previously screened for its antifungal activity against *Candida* species, *Cryptococcus* species, and chromoblastomycosis agents [14–17]. Thus, the purpose of this study was to screen this collection in search of compounds with potential anti-*S*. *brasiliensis* and *S*. *schenckii* activity.

## Materials and methods

### Microorganisms

The reference isolates *S*. *brasiliensis* CBS 133006 and *S*. *schenckii* ATCC 32286 were used in this study. In addition, eight clinical isolates, six from human sporotrichosis patients (*S*. *brasiliensis* B972 and HE06; and *S*. *schenckii* Ss22, Ss42, Ss73, and Ss110) and two from felines (*S*. *brasiliensis*: Ss53 and Ss245) were used [18]. All isolates were stored in saline solution containing 10% glycerol and 10% glucose at -20°C. The filamentous form was cultivated for 7 days in Sabouraud broth (Difco, United States), with orbital shaking (at 150 rpm), at 36°C. Then, an aliquot containing 10^5^ CFU/ml was inoculated into brain heart infusion broth (Difco, USA) supplemented with 2% glucose (pH 7.8) and cultivated at 36°C, with orbital shaking for 7 days, in order to obtain yeast cells. All assays were carried out using the yeast form as it is the pathogenic form.

### Compounds

The Pathogen Box library was obtained from Medicines for Malaria Venture (MMV, Switzerland) and contained 400 compounds diluted in dimethyl sulfoxide (DMSO) at 10 mM. A stock solution of all compounds was prepared in DMSO at 1 mM and stored at -20°C. Itraconazole (Sigma Chemical Co., USA) was used as a reference antifungal, and a stock solution (1 mM in DMSO) was kept at -20°C.

### Screening the compound library for inhibition of *Sporothrix* spp. growth

The reference isolates *S*. *brasiliensis* CBS 133006 and *S*. *schenckii* ATCC 32286 were used to screen The Pathogen Box compounds for putative inhibitors of fungal growth according to the following protocol: dx.doi.org/10.17504/protocols.io.bje9kjh6. The final concentration of compounds was 1 μM following the MMV recommended guidelines for screening (https://www.mmv.org/mmv-open/pathogen-box/pathogen-box-supporting-information), while the final concentration of fungal cells was 1 x 10^5^ CFU/ml. Itraconazole (reference antifungal) was also tested at 1 μM. After 48 h (at 35°C in a 5% CO_2_ atmosphere), fungal growth was analyzed by visual inspection in an inverted light microscope and quantified by spectrophotometric readings. Inhibitions of more than 80% were defined as the cut-off to select for the most promising compounds, corresponding to clearly visible prevention of growth when samples were initially analyzed by visual inspection. Experiments were performed in quadruplicate.

### Determination of minimum inhibitory concentration values (MICs) against *S*. *brasiliensis* and *S*. *schenckii*

The MIC of the most active compounds were determined using the *in vitro* broth microdilution technique described by the CLSI [19] with modifications according to the following protocol: dx.doi.org/10.17504/protocols.io.bjgxkjxn. MIC values were compared with that of the reference drug itraconazole. Yeasts (1 x 10^5^ CFU/ml) were treated with different concentrations of compounds (0.002–1 μM) for 48 h, at 35°C, in a 5% CO_2_ atmosphere. Fungal growth was analyzed by visual inspection in an inverted light microscope and quantified by spectrophotometric readings. Concentrations that inhibit 50% and 80% of fungal growth (IC_50_ and IC_80_, respectively) were estimated. Results are presented as the mean of two independent experiments, performed in duplicate.

### Cytotoxicity assays

Cytotoxicity assays with MMV102872 and iodoquinol (selected as the most promising compounds) were performed using a mammalian epithelial cell line (LLC-MK_2_; ATCC CCL-7) according to the following protocol: dx.doi.org/10.17504/protocols.io.bjgbkjsn. Several concentrations of compounds (0.1–10 μM) were tested, concentrations that elicited 50% cytotoxicity (CC_50_) were estimated, and the selectivity towards *Sporothrix* spp. was determined using the IC_80_ values obtained previously. The selectivity index (SI) of compounds was calculated using the following equation: SI = CC_50_/ IC_80_ median. Results are representative of two independent experiments, performed in triplicate.

### Treatment of fungal cells

The effect of MMV102872 and iodoquinol (MMV002817) on the morphology and metabolism of *S*. *brasiliensis* yeasts (CBS133006) was evaluated by scanning electron microscopy and flow cytometry. Standardized yeast suspensions (1 x 10^5^ CFU/ml) were treated with the IC_50_ concentrations (0.25 μM) of MMV102872 or iodoquinol (MMV002817) in supplemented RPMI, for 48 h, at 36°C, with orbital shaking. Untreated controls were grown in the absence of drugs in parallel. Next, samples were centrifuged at 927 G for 3 min, washed three times in sterile PBS and processed for scanning electron microscopy or flow cytometry as described below.

### Scanning electron microscopy

Untreated and treated cells (obtained as described in “Treatment of fungal cells”) were fixed in 2.5% glutaraldehyde and 4% formaldehyde, in 0.1 M cacodylate buffer, for 1 h and then washed three times in 0.1 M cacodylate buffer. Samples were adhered to poly-L-lysine-coated glass coverslips, dehydrated in a graded ethanol series, critical-point-dried in CO_2_, and coated with gold. Images were obtained in a ZEISS EVO 10 scanning electron microscope (ZEISS Company, Germany) and processed using Photoshop software (Adobe, USA). The area distribution of 100 cells was measured and the count of damaged cells was performed using ImageJ software (National Institute of Health, USA).

### Flow cytometry

Neutral lipid accumulation was evaluated by flow cytometry using the fluorescent marker BODIPY 493/503 (Thermo Fisher Scientific, USA). Plasma membrane integrity was evaluated using SYTOX Green (Thermo Fisher Scientific, USA), which is impermeable to intact membranes, but penetrates compromised membranes and stains nucleic acids. Untreated and treated cells (obtained as described in “Treatment of fungal cells”) were incubated with 200 μl containing 20 μM of each fluorochrome for 30 min at room temperature in the dark. Cells were washed in PBS, lightly fixed in 2% formaldehyde, and washed again in PBS. The fluorescence intensity of stained cell populations was quantified in a BD Accuri C6 flow cytometer (BD Biosciences, USA) that counted 10,000 or 2000 events per sample for BODIPY 493/503 or SYTOX Green, respectively. Data were analyzed using BD Accuri C6 software. Experiments were repeated three times. Data depicted in the results section shows the mean and standard error of the mean of one representative experiment.

### Statistical analyses

Statistical analysis was performed using Prism 6.01 software (GraphPad Software, USA), by one-way ANOVA (with Dunnett’s *post hoc* test). Statistical significance was considered when p < 0.05.

## Results

### Screening of the Pathogen Box library identified five potential *Sporothrix* growth inhibitors

**Fig 1:**
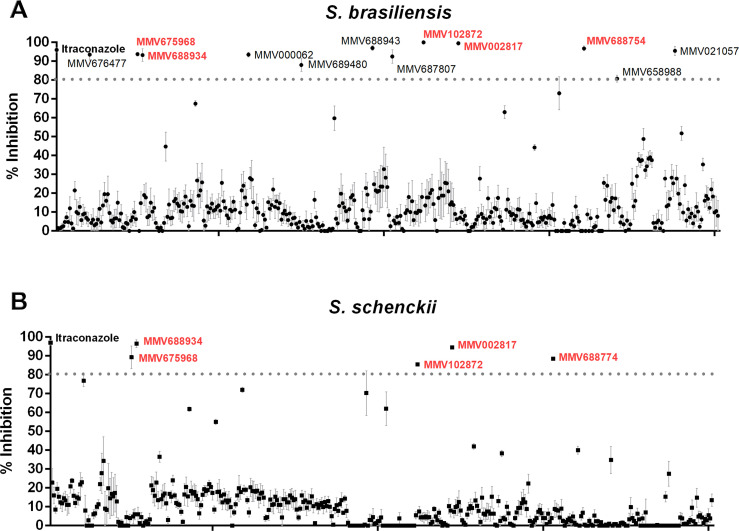
High-content screening of the Pathogen Box library. Percentage of growth inhibition of (A) *Sporothrix brasiliensis* and (B) *Sporothrix schenckii* after exposure to 1 μM of the library compounds for 48 hours. Data represent the mean ± SEM and are fully described in S1 Table.

The initial screen of the 400 compounds in the Pathogen Box identified 12 compounds that inhibited *S*. *brasiliensis* CBS 133006 growth by more than 80% (**Fig 1A**). These compounds are (i) the antifungal drug posaconazole (MMV688754); (ii) the insecticide tolfenpyrad (MMV688934); (iii) the agricultural fungicides difenoconazole and azoxystrobin (MMV688943 and MMV021057); (iv) three commercial drugs used to treat different diseases (MMV002817 –iodoquinol, MMV000062 –pentamidine, and MMV689480 –buparvaquone); and (v) five new molecules (MMV675968, MMV102872, MMV676477, MMV687807, and MMV658988) (**Fig 1A**). In contrast, only five compounds were able to inhibit the growth of *S*. *schenckii* ATCC 32286 by more than 80% (**Fig 1B**). Interestingly, all those five compounds were also able to inhibit *S*. *brasiliensis* growth (MMV688754, MMV688934, MMV002817, MMV675968, and MMV102872).

Among the five compounds that are active for both species, compounds MMV688754 and MMV688934 were excluded from subsequent experiments as they are commercial antifungal drugs (MMV688754-posaconazol) and an agrochemical insecticide (MMV688934-tolfenpyrad). Therefore, MMV675968, MMV102872, and MMV002817 (iodoquinol) were selected for further studies exploring their potential antifungal activity and targets.

### Antifungal activity of MMV675968, MMV102872, and MMV002817 (iodoquinol) against *Sporothrix* spp. isolates

**Fig 2:**
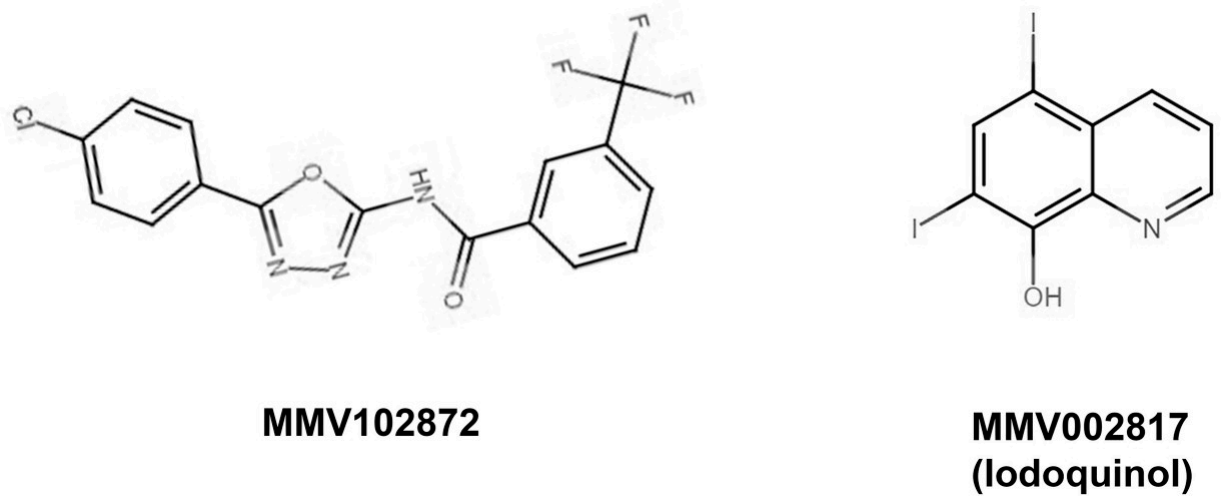
Molecular structure of MMV102872 and iodoquinol (MMV002817).

MMV675968, MMV102872, and iodoquinol were able to inhibit 80% of *S*. *brasiliensis* growth similarly to itraconazole–i.e. similar IC_80_ values (**Table 1**); while only MMV102872 had a similar inhibitory effect against *S*. *schenckii* isolates (no statistical significance compared with itraconazole IC_80_) (**Table 1**). Thus, MMV102872 and iodoquinol were the most active compounds against *S*. *brasiliensis* and *S*. *schenckii* (**Fig 2**).

**Table 1:** Susceptibility profile of *Sporothrix* isolates to MMV675968, MMV102872, and iodoquinol.

Isolates	Itraconazole		MMV675968		MMV102872		MMV002817	
							(Iodoquinol)	
***S*. *brasiliensis***	**IC**_**50**_	**IC**_**80**_	**IC**_**50**_	**IC**_**80**_	**IC**_**50**_	**IC**_**80**_	**IC**_**50**_	**IC**_**80**_
Reference isolate								
CBS133006	0.03	0.25	0.25	0.5	0.25	0.5	0.25	0.5
Human isolates								
B972	ND	0.25	0.5	1	0.25	0.5	0.25	0.5
HE06	ND	0.25	0.125	0.5	0.125	0.25	0.25	0.5
Feline-borne isolates								
Ss53	0.25	0.5	0.06	0.25	0.125	0.25	0.25	0.5
Ss245	0.06	0.25	0.125	0.5	0.125	0.25	0.25	0.5
**Median**	**0.06**	**0.25**	**0.125**	**0.5**	**0.125**	**0.25**	**0.25**	**0.5**
***S*. *schenckii***	**IC**_**50**_	**IC**_**80**_	**IC**_**50**_	**IC**_**80**_	**IC**_**50**_	**IC**_**80**_	**IC**_**50**_	**IC**_**80**_
Reference isolate								
ATCC32286	0.125	0.25	0.03	0.125	ND	0.25	0.5	1
Human isolates								
Ss22	0.125	0.25	>1	>1	0.25	0.5	0.5	1
Ss42	ND	0.25	ND	1	ND	0.25	0.25	1
Ss73	0.06	0.25	1	>1	0.125	0.25	0.25	0.5
Ss110	0.03	0.125	0.5	1	0.125	0.25	0.25	0.5
**Median**	0.09	0.25	0.5	1	0.125	0.25	0.25	1

Minimum inhibitory concentration of different human and feline isolates of *S*. *schenckii* and *S*. *brasiliensis* to MMV675968, MMV102872, and Iodoquinol, compared with itraconazole.

All values are expressed in μM.

IC_50_, concentration that inhibits 50% of fungal growth. IC_80_, concentration that inhibits 80% of fungal growth.

ND, Not determined due to the detection limit of the microdilution technique.

### MMV102872 and iodoquinol showed low cytotoxicity against human cell lines *in vitro*

The CC_50_ of MMV102872 and iodoquinol using the mammalian cell line LLC-MK_2_ were 8.7 μM and 5 μM, respectively, resulting in high selectivity index of each compound to both *S*. *brasiliensis* and *S*. *schenckii* (**Table 2**).

**Table 2:** Selectivity of compounds towards *Sporothrix* spp.

Compounds	IC_80_ medians (μM)		Cytotoxicity against LLC-MK_2_ cells	Selectivity Index^a^	
	*S*. *brasiliensis*	*S*. *schenckii*	CC_50_ (μM)	*S*. *brasiliensis*	*S*. *schenckii*
MMV102872	0.25	0.25	8.7	34.8	34.8
MMV002817 (iodoquinol)	0.5	1	5	10	5

Cytotoxicity to mammalian cells *in vitro* and selectivity of MMV102872 and iodoquinol towards *Sporothrix brasiliensis* and *Sporothrix schenckii* cells.

^a^ Selectivity index (SI) was defined as SI = CC_50_/IC_80_ median.

### MMV102872 and iodoquinol altered fungal cell morphology and lipid metabolism and disrupted cell membrane integrity

**Fig 3:**
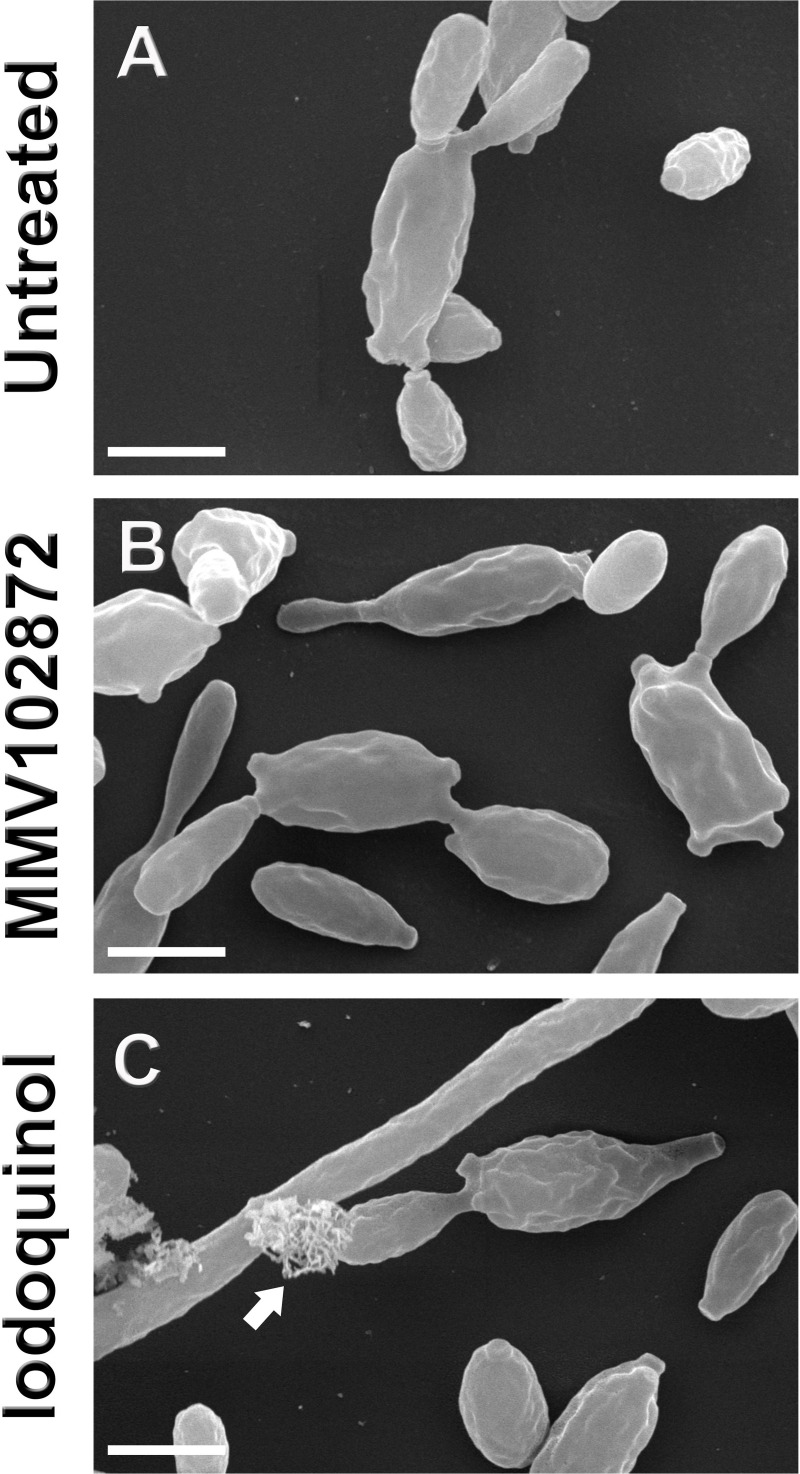
Effect of MMV102872 and iodoquinol (MMV002817) treatment on *Sporothrix brasiliensis* cell morphology. Fungal cells were exposed to the IC_50_ of compounds (0.25 μM) for 48 h and scanning electron microscopy images showed cell disruption and overflow of intracellular contents induced by iodoquinol treatment (C, arrow). Scale bars: 5 μm.

**Fig 4:**
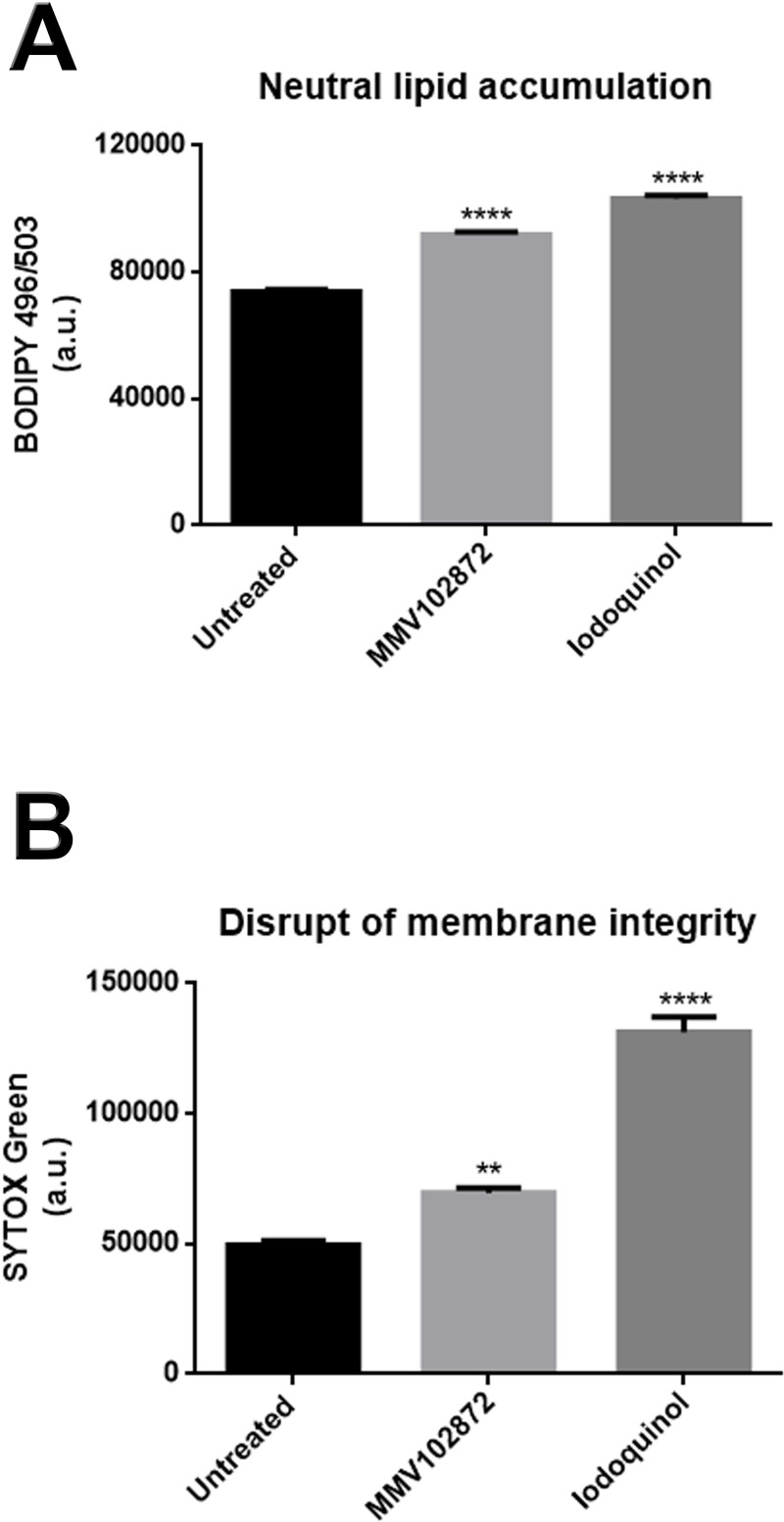
Levels of neutral lipids and damage to the plasma membrane in *Sporothrix brasiliensis* treated with MMV102872 and iodoquinol (MMV002817). (A) Neutral lipid accumulation was observed after treatment with the IC_50_ (0.25 μM) of either compound for 48 h, as revealed by accumulation of BODIPY 496/503 stain. (B) SYTOX Green labelling revealed that both treatments induced damage to the plasma membrane, which was even more pronounced after iodoquinol treatment. **p < 0.01 and ****p < 0.0001.

The effects of MMV102872 and iodoquinol were evaluated against *S*. *brasiliensis* as a representant of genus *Sporothrix*. *S*. *brasiliensis* was selected based on its epidemiological importance in Brazil, where its associated with endemic transmission and also unusual clinical presentations of human sporotrichosis. Scanning electron microscopy analysis of *S*. *brasiliensis* (CBS 133006) revealed the presence of disrupted cells and overflow of intracellular contents after exposure to iodoquinol (**Fig 3C**, arrow), which was not observed for untreated or MMV102872 treated cells (**Table 3**). Moreover, treatments with MMV102872 and iodoquinol induced an increase in cell size of 7% and 17%, respectively (**Table 3**). Flow cytometry results revealed that MMV102872 and Iodoquinol induced significant accumulation of neutral lipids and disruption of the plasma membrane integrity; being such cellular alterations more pronounced after treatment with iodoquinol (**Fig 4**).

**Table 3:** Cell size of *Sporothrix brasiliensis* and damage cells after treatments.

	Mean area (μm^2^)	Disrupted cells
**Untreated**	2.9	0%
**MMV102872**	3.1	0%
**Iodoquinol**	3.4 **	8%

Quantification of the area and disrupted cells performed based on scanning electron microscopy images.

**p < 0.01 vs. untreated by one-way ANOVA and Dunnett’s test.

## Discussion

Over the last 50 years, several fungal infections have emerged as important public health problems [20]. In the present work, we screened the Pathogen Box library in search of promising molecules with anti-*Sporothrix* activity, showing that 3% (12/400) and 1.25% (5/400) of them were able to inhibit *S*. *brasiliensis* and *S*. *schenckii*, respectively.

*S*. *brasiliensis* and *S*. *schenckii* can exhibit distinct *in vitro* susceptibility profiles to commercial antifungals [21]. Besides, higher rates of *in vivo* resistance, as wells as prolonged treatment in sporotrichosis human cases, are reported in *S*. *schenckii* infections [8]. We observed that *S*. *brasiliensis* was susceptible to a higher number of compounds from the library, compared with *S*. *schenckii*. The lower sensitivity of *S*. *schenckii* to the compounds could be in part related to differences in the structure and composition of the cell wall of this species compared to *S*. *brasiliensis* [22].

Three compounds that showed activity against *S*. *brasiliensis* in the initial screening are usually used as agrochemicals: difenoconazole (MMV688943), azoxystrobin (MMV021057), and tolfenpyrad (MMV688934), the last one also being effective against *S*. *schenckii*. The expanding use of antifungal-based agrochemicals has been correlated with the emergence of antifungal resistance among pathogenic fungi [23], and *Sporothrix* spp. are most often found in the environment, which increases the probability of exposure to these compounds, ultimately reducing its susceptibility to them. Therefore, as our goal is to treat humans and animals, we decided to exclude those agrochemical molecules from our pool of candidates for the development of new human and/or animal antifungal agents. However, it is important to emphasize that the use of some of those compounds (mainly tolfenpyrad) could be explored for decontamination of environmental areas with high fungal loads that may be a focus of animal/human infection [6].

The initial screening also showed that posaconazole (MMV688754) and three other commercial drugs were able to inhibit *S*. *brasiliensis* growth: pentamidine (MMV000062), buparvaquone (MMV689480), and iodoquinol (MMV002817); however, only posaconazole (MMV688754) and iodoquinol (MMV002817) were also effective against *S*. *schenckii* (**Fig 1**). The *in vitro* and *in vivo* activity of posaconazole against *Sporothrix* spp. is well known [21, 24]; however, its high costs compromises usage in lower-income countries of South America, like Brazil, where sporotrichosis also figures as a higly prevalent mycosis.

Among the new molecules, five compounds (MMV675968, MMV102872, MMV676477, MMV687807, and MMV658988) exhibited high activity against *S*. *brasiliensis*; however, only MMV675968 and MMV102872 were also effective against *S*. *schenckii* (**Fig 1**). The new compounds that showed anti-*Sporothrix* activity are diverse, and the previously described mechanisms of action for several cell types and microorganisms are summarized in **Table 4**. The identification of five new molecules with possibly distinct cellular targets opens perspectives for future studies, because these molecules have a different mechanism of action than antifungals currently available to treat fungal infections, a relevant discovery due to the increase in fungus resistance [23].

**Table 4:** New compounds with anti-*Sporothrix* activity.

Compound	Class	Known antimicrobial activity	Described in this work	Mechanism of action
MMV676477	pyrimidinone-pyrazole	*Mycobacterium tuberculosis* [13]	*S*. *brasiliensis*	tyrosine kinase inhibitor [13]
MMV658988	pyrimidine	*Trypanosoma* and *Leishmania* species [13]	*S*. *brasiliensis*	methionine aminopeptidases inhibitor [13]
MMV687807	hydroxybenzamide	*Mycobacterium tuberculosis*, *Candida albicans* [13, 14]	*S*. *brasiliensis*	disruption of the mitochondrial proton gradient [13]
MMV675968	quinazoline	*Toxoplasma gondii*, *Cryptosporidium parvum*, *Pneumocystis jrovecii*, *Candida albicans* [13, 14]	*S*. *schenckii*, *S*. *brasiliensis*	dihydrofolate reductase inhibitor [13]
MMV102872	oxadiazole	*Mycobacterium tuberculosis*, *Staphylococcus aureus*, *Candida auris* [13, 16]	*S*. *schenckii*, *S*. *brasiliensis*	unknown

Summary of chemical and biological information of five new compounds with antifungal activity against *Sporothrix* spp. described in this study.

According to the above-cited results from the initial susceptibility assays, iodoquinol (MMV002817) and the new molecules MMV675968 and MMV102872 were selected for further evaluation as anti-*Sporothrix* agents. Iodoquinol (MMV002817) is a quinoline derivative used as an antiparasitic drug for intestinal worms (also known as diiodohydroxyquinoline) (**Fig 2**) [25]. MIC values showed that iodoquinol is highly active against *Sporothrix* spp. at concentrations as low as 1 μM, inducing almost complete inibition of proliferation (IC_80_) of all 10 clinical isolates tested (**Table 1**). Iodoquinol has also been reported to inhibit *in vitro* growth of *Candida* species and chromoblastomycosis agents [16, 17]. The IC_50_ for planktonic cells of *Candida* spp. was similar to what we described here for *Sporothrix* spp. (0.25 μg/ml–corresponding to 0.63 μM), and growth inhibition of 100% of chromoblastomycosis agents was observed with 1.25 μM iodoquinol [16, 17]. Therefore, a comprehensive study including a panel of diverse fungi would be of interest to elucidate whether iodoquinol has a broad spectrum of antifungal activity.

Compound MMV675968 (5-chloro-6-[(2,5-dimethoxyanilino) methyl] quinazoline-2,4-diamine) was designed as a dihydrofolate reductase inhibitor, an enzyme conserved in prokaryotes and eukaryotes that catalyzes the NADPH-dependent reduction of dihydrofolate into tetrahydrofolate [13]. Previous studies demonstrated that MMV675968 also exhibited an inhibitory effect against some protozoa (*Toxoplasma gondii* and *Cryptosporidium parvum*) and fungi (*Pneumocystis jrovecii* and *Candida albicans*) [13, 14] (**Table 4**). Our results showed that although MMV675968 presented high activity against *S*. *brasiliensis*, it was less effective at preventing proliferation of *S*. *schenckii* at concentrations lower than 1 μM (**Table 1**). The IC_50_ of MMV675968 against planktonic cells of *C*. *albicans* was also higher than 1 μM (3.12 μM), and inhibition of biofilms was only achieved at 50 μM [14].

Finally, MMV102872 (*N*-[5-(4-chlorophenyl)-1,3,4-oxadiazol-2-yl]-3-(trifluoromethyl) benzamide) is an azole derivative (**Fig 2**) with previously reported antimycobacterial and anti-*Staphylococcus* activities [13]. Our data revealed that *Sporothrix* spp. was more susceptible to MMV102872 than to MMV675968, being able to prevent fungal growth of both *Sporothrix* species at concentrations lower than 0.5 μM, similarly to itraconazole (**Table 1**). Wall and co-workers recently showed that, at 20 μM, MMV102872 inhibits 88% of growth in the emerging pathogen *Candida auris* [16]. The anti-*Sporothrix* activity observed was remarkably higher than that observed for *C*. *auris*.

Despite its demonstrated antimicrobial activities, no mechanism of action has been reported for MMV102872 in the literature, while the amoebicidal activity of iodoquinol has been associated with its ability to chelate iron [13]. MMV102872 is an azole-derivative, and previous reports have shown that inhibition of ergosterol biosynthesis by azoles leads to accumulation of neutral lipids; therefore, the accumulation of neutral lipids was quantified in *Sporothrix*-treated and non-treated cells, as well as cell membrane integrity effects given its central role in maintaining cellular homeostasis [26]. Disruption in the membrane was observed to a lesser extent after treatment with MMV102872, as well as neutral lipid accumulation, when compared to iodoquinol (**Fig 4**). Since this compound is an azole-derivative, we speculate that, in *Sporothrix* spp., MMV102872 may be interfering with ergosterol biosynthesis [26]. Alterations in iron absorption could disrupt enzymatic activity and metabolic pathways, culminating in irreversible cell damage and rupture, as observed by SEM in *Sporothrix* cells after treatment with iodoquinol (**Fig 3C**). However, further studies are needed to elucidate the mechanism(-s) involved in the antifungal activity exerted by MMV102872 and iodoquinol.

Importantly, the cytotoxicity results reported here demonstrate that MMV102872 and iodoquinol are more selective towards fungi than mammalian cells (**Table 2**). Previous reports using HepG2 cells showed a CC_50_ for MMV102872 of 9 μM; and a CC_20_ for iodoquinol of 2.5 μM (reported in Pathogen Box documents: https://www.mmv.org/mmv-open/pathogen-box/about-pathogen-box#composition). Together, these data suggest that both compounds have reduced toxicity and are potential candidates to advance to *in vivo* and clinical studies.

Iodoquinol has been used for the treatment of protozoal infections of the gastrointestinal tract; however, it is not able to penetrate into the intestinal epithelium [25]. Topical formulations containing 1% iodoquinol are available to treat dermatoses and exhibit antifungal and antibacterial properties [27] and could be repurposed as an adjuvant topical treatment for sporotrichosis in association with oral itraconazole therapy. Thus, future *in vivo* and clinical studies are required to evaluate the effectiveness of iodoquinol in the treatment of sporotrichosis.

## Conclusion

In this work we screened the Pathogen Box library and identified two compounds, MMV102872 and iodoquinol, with promising anti-*Sporothrix* activity. These compounds were effective against both *Sporothrix* species endemic in Latin America: *S*. *schenkii* and *S*. *brasiliensis* and demonstrated reduced toxicity to mammalian cell lines *in vitro*. Mechanistic studies indicated that both compounds induced neutral lipid accumulation and disruptions in cell membrane homeostasis. Finally, based on the described results we suggest that MMV102872 and iodoquinol are promising molecules and could be used as a scaffold for the development of new antifungal agents.

## Supporting information

S1 Table Percentage of growth inhibition at 1 μM. Inhibition of *Sporothrix brasiliensis* CBS 133006 and *Sporothrix schenckii* ATCC 32286 after exposure to 1 μM of the library compounds for 48 hours.
(XLSX)
